# Prion type 2 selection in sporadic Creutzfeldt–Jakob disease affecting peripheral ganglia

**DOI:** 10.1186/s40478-021-01286-4

**Published:** 2021-11-24

**Authors:** Anna Hofmann, Arne Wrede, Wiebke M. Jürgens-Wemheuer, Walter J. Schulz-Schaeffer

**Affiliations:** 1 Prion and Dementia Research Unit, Institute of Neuropathology, University Medical Center, Göttingen, Germany; 2 Department of Neurodegenerative Diseases, Hertie Institute for Clinical Brain Research, University Hospital Tübingen, Tübingen, Germany; 3 German Center for Neurodegenerative Diseases (DZNE), University of Tübingen, Tübingen, Germany; 4 Institute of Neuropathology, Medical Faculty, Saarland University, Building 90.3, 66421 Homburg/Saar, Germany

**Keywords:** Transmissible spongiform encephalopathies, Protein aggregation disease, Celiac ganglion, Sympathetic trunk ganglia, PET-blot

## Abstract

In sporadic Creutzfeldt–Jakob disease (sCJD), the pathological changes appear to be restricted to the central nervous system. Only involvement of the trigeminal ganglion is widely accepted. The present study systematically examined the involvement of peripheral ganglia in sCJD utilizing the currently most sensitive technique for detecting prions in tissue morphologically. The trigeminal, nodose, stellate, and celiac ganglia, as well as ganglia of the cervical, thoracic and lumbar sympathetic trunk of 40 patients were analyzed with the paraffin-embedded tissue (PET)-blot method. Apart from the trigeminal ganglion, which contained protein aggregates in five of 19 prion type 1 patients, evidence of prion protein aggregation was only found in patients associated with type 2 prions. With the PET-blot, aggregates of prion protein type 2 were found in all trigeminal (17/17), in some nodose (5 of 7) and thoracic (3 of 6) ganglia, as well as in a few celiac (4 of 19) and lumbar (1 of 5) ganglia of sCJD patients. Whereas aggregates of both prion types may spread to dorsal root ganglia, more CNS-distant ganglia seem to be only involved in patients accumulating prion type 2. Whether the prion type association is due to selection by prion type-dependent replication, or due to a prion type-dependent property of axonal spread remains to be resolved in further studies.

**Supplementary Information:**

The online version contains supplementary material available at 10.1186/s40478-021-01286-4.

## Introduction

Prion diseases or transmissible spongiform encephalopathies are neurodegenerative disorders and always fatal. The infectious agent seems to be an unconventional one as it appears that the pathologic misfolded form (PrP^Sc^) of a physiological protein (PrP^C^) is responsible for its transmissibility. In 1982, Stanley Prusiner therefore coined the term “proteinaceous infectious particle” and its abbreviation “prion” [30]. With an incidence of 1–2 in 1,000,000, Creutzfeldt–Jakob disease (CJD) is the most common prion disease in humans. Most CJD cases occur sporadically, but there are also familial and acquired forms that occur less frequently [39]. Acquired CJD includes iatrogenic forms, as well as the so-called ‘variant’ CJD (vCJD), which is most likely caused by oral transmission of the bovine spongiform encephalopathy (BSE), a prion disease in cattle [7, 40].

Several animal models with orally transmitted prion diseases reveal an involvement of the peripheral nervous system, mainly of peripheral ganglia. Particularly the orally scrapie-challenged hamster model gave insights into the temporo-spatial spread of agents initiating from enteric regions, i.e. the myenteric and submucosal plexuses of the ileum [24]. The authors later described the existence of two centripetal anatomical routes, where pathological prion protein was first detectable in autonomic structures and later in sensory ones. The latter finding was interpreted as a secondary centrifugal spread to sensory organs. In the splanchnic nerve circuitry, the agent was detected in the celiac and mesenteric ganglion complex at 56 days post infection (dpi) and in the intermediolateral cell column of the thoracic spinal cord at 69 dpi, followed by the dorsal root ganglion at 76 dpi [4, 20]. Similarly, in the vagus nerve circuitry PrP^Sc^ was detected in the dorsal motor nucleus of the vagus in the brainstem (62 dpi) and later in the nodose ganglion (90 dpi) [3, 5]. On the other hand, Holznagel et al*.* (2015) demonstrated in their non-human primate model of oral infection in Cynomolgus macaques that after entering the enteric nervous system the prions invade the central nervous system via sensory afferent tracts at the dorsal root entry zone of lumbar spinal cord segments. In the following, they spread in a centrifugal fashion via the anterior horn along preganglionic fibers into sympathetic ganglia [17].

Among the human prion diseases, an involvement of peripheral tissues is best established for the orally transmitted vCJD. A pathogenic involvement of various lymphatic organs has been described here [13–15]. Pathologic prion protein accumulations have been detected in sympathetic ganglia of vCJD but not of sCJD patients [11, 12]. An involvement of trigeminal ganglia has been demonstrated in sCJD, and is thought to be caused by a centrifugal spread of the disease over time [22].

The aim of this study was to search for prion aggregates in the autonomous nervous system of patients with *sporadic* CJD. We performed a histological examination of peripheral ganglia collected during autopsy from patients with neuropathologically confirmed sCJD. It is well known that the clinical characteristics of sCJD are generally determined by the underlying polymorphism in codon 129 of the patient’s PrP (methionine versus valine), as well as by the causative PrP^Sc^ type (i.e. 1 vs. 2), which differ in their protein conformation. The combination *PRNP*-codon 129-polymorphism and prion type defines six variants of sCJD, i.e. MM1, MM2, MV1, MV2, VV1 and VV2. We analyzed our histological data with a particular focus on this classification [27]. The pattern of autonomous nervous system involvement may be helpful in the discussion of the disease spread.

## Materials and methods

### Specimens and tissue preparation

We analyzed tissues from 40 sCJD patients with a confirmed *post mortem* diagnosis. Regarding the combination of the methionine (M) valine (V) polymorphism at codon 129 and the prion type, our patient cohort included 18 MM1, 9 VV2, 11 MV2 (displaying Kuru plaques and in 9 out of 11 patients confluent vacuoles in the cortex), one MV1 and one MM2.

According to the availability of the respective tissues, we examined the trigeminal ganglion in 36, the nodose ganglion in 16, the stellate ganglion in 20, the ganglia of the cervical sympathetic trunk in 15, the thoracic sympathetic trunk ganglia in eleven, the pelvic plexus in nine and the celiac ganglion in 35 sCJD patients. The evaluation of samples as either `positive´ or `negative´ was followed by a semiquantitative assessment of staining intensity as `weakly´, `moderately´ or `strongly´ positive (Table 1). As negative controls we chose four patients with a prion-independent CNS disease: two with Alzheimer`s disease (one genetic AD), one with frontotemporal lobar degeneration, and one with a viral encephalitis (Table 1).

**Table 1:** Sporadic CJD and control patients analyzed with the PET-blot method including semiquantitative organ assessment

Case no.	Codon 129 plymprphism	Prion type/diagnosis	Trigeminal ganglion	Nodose ganglion	Cervical sympathetic trunk	Stellate ganglion	Thoracic sympathetic trunk	Celiac ganglion	Lumbal sympathetic trunk
1	MM	1	+	n.a	n.a	n.a	n.a	–	n.a
2	MM	1	–	n.a	n.a	n.a	n.a	–	n.a
3	MM	1	–	n.a	n.a	n.a	n.a	–	n.a
4	MM	1	++	n.a	n.a	n.a	n.a	n.a	n.a
5	MM	1	–	n.a	n.a	n.a	n.a	–	n.a
6	MM	1	–	n.a	n.a	n.a	n.a	n.a	n.a
7	MM	1	–	n.a	n.a	n.a	n.a	–	n.a
8	MM	1	–	–	n.a	–	n.a	–	n.a
9	MM	1	–	–	n.a	–	n.a	–	n.a
10	MM	1	+	–	–	–	n.a	n.a	n.a
11	MM	1	–	–	–	–	n.a	–	n.a
12	MM	1	–	n.a	––	–	n.a	–	n.a
13	MM	1	–	–	n.a	–	–	–	n.a
14	MM	1	–	–	–	–	–	–	n.a
15	MM	1	+	n.a	–	–	–	–	–
16	MM	1	+	–	–	–	–	–	–
17	MM	1	–	–	–	–	–	–	–
18	MM	1	–	–	–	–	n.a	–	–
19	MM	2	n.a	n.a	n.a	n.a	n.a	–	n.a
20	MV	1	–	n.a	n.a	n.a	n.a	–	n.a
21	MV	2	n.a	n.a	n.a	n.a	n.a	+++	n.a
22	MV	2	n.a	n.a	n.a	n.a	n.a	–	n.a
23	MV*	2	++	n.a	n.a	n.a	n.a	–	n.a
24	MV	2	+++	n.a	n.a	n.a	n.a	++	n.a
25	MV	2	+	n.a	n.a	n.a	n.a	–	n.a
26	MV	2	+	n.a	n.a	n.a	n.a	–	n.a
27	MV	2	+	n.a	n.a	n.a	n.a	–	n.a
28	MV	2	++	+	n.a	–	n.a	n.a	n.a
29	MV*	2	+++	n.a	–	–	–	+++	n.a
30	MV	2	+++	n.a	–	–	+++	–	–
31	MV	2	+++	++	–	–	+++	–	–
32	VV	2	++	n.a	n.a	n.a	n.a	–	n.a
33	VV	2	+++	n.a	n.a	n.a	n.a	–	n.a
34	VV	2	+++	n.a	n.a	n.a	n.a	–	n.a
35	VV	2	+++	n.a	n.a	n.a	n.a	–	n.a
36	VV	2	+++	+++	–	–	n.a	+	++
37	VV	2	n.a	++	n.a	–	n.a	n.a	n.a
38	VV	2	++	–	–	–	–	–	n.a
39	VV	2	+++	+++	–	–	++	–	–
40	VV	2	+++	–	–	–	–	–	–
41	MM	genet. AD	–	n.a	n.a	n.a	n.a	–	n.a
42	MV	AD	–	–	–	–	n.a	–	n.a
43	MV	FTLD	–	–	n.a	–	n.a	–	–
44	n.a	viral encephalitis	–	–	n.a	–	–	–	–

The staining reactions were rated as strongly (+++), moderately (++) and weakly (+) positive or negative (–). ‘n.a.’ indicates that the corresponding organ has not been available for investigation. AD = Alzheimer's Disease, FTLD = frontotemporal lobal degeneration. MV2* indicates *PRNP*-codon 129 heterozygous prion type 2-patients that did not show confluent vacuoles in the cortex

Formalin-fixed tissues were sectioned and put into cassettes where they were decontaminated in 99% concentrated formic acid for one hour. After a second fixation period in 4% buffered formaldehyde, the tissues were embedded in paraffin (Paraplast plus, *McCormick scientific*), using isopropanol instead of ethanol during the dehydrating process. 1-3 µm sections were cut with a microtome (*Leica, SM2000R*), transferred to glass slides or nitrocellulose membranes (0.45 µm pore size, *Bio-Rad*), and dried at 56 °C for 12 h (glass slides) or 48 h (membranes).

### Paraffin-embedded tissue (PET)-blot

The paraffin-embedded tissue blot (PET-blot) is the most sensitive technique for the detection of protein aggregates in tissue sections and was carried out as described before [31]. In brief, the membranes were rehydrated (again using isopropanol instead of ethanol) and the tissues were digested overnight with proteinase K (*Sigma-Aldrich*) at 56 °C with an enzyme concentration of 250 µg/ml to remove all non-aggregated proteins. After washing in TBST (10 mM Tris, 100 mM NaCl, 0.05% Tween 20 in aqua bidest.), they were denatured in 4 M GdnSCN (*Amresco*), repeatedly washed in TBST, and blocked in 0.2% casein (I-block, *Applied Biosystems*, 1 ml Tween 20 in 1 l phosphate-buffered saline, PBS). This was followed by incubation with the 1:5000 diluted primary monoclonal antibody 12F10 (kindly provided by Prof. Dr. Bodemer, *German Primate Center*, Göttingen, Germany) for 90 min, washing in TBST and incubation with the 1:1000 diluted alkaline phosphatase (AP)-coupled secondary goat anti-mouse antibody (*Dako*) for 60 min. After washing in TBST and NTM (100 mM Tris pH 9.5, 100 mM NaCl, 50 mM MgCl_2_ in distilled water) a formazan-reaction (90 µl nitro-blue tetrazolium, *Sigma-Aldrich*, 66 µl bromo-chloro-indolylphosphate, *Boeringer-Mannheim* in 20 ml NTM) was used to visualize the antibody binding. The dyeing reaction was stopped with PBS once the positive control had reached an adequate signal, generally after about 15 min. After washing in demineralized water and adding 0.5 M EDTA to fix the stain, membranes were dried overnight and subsequently examined under a reflected-light microscope (*Olympus*). Serial sections of all specimens stained with hematoxylin and eosin aided the identification of anatomical details in PET-blot slides.

We confirmed our PET-blot results with the primary antibodies 3F4 (1:2000; kindly provided by PD Dr. Beekes, *Robert Koch Institute,* Berlin, Germany) and ICSM-18 (*1:5000 - 1:10.000; D-Gen*), even though the staining pattern obtained with 12F10 was most distinct and intense. The latter, therefore, represented our standard for evaluating the presence of prion aggregates and was the basis for the results presented in this publication. In Additional file 1: Fig. 1 we show the nodose ganglion from a negative control (#43) stained with mAb 12F10 (C) in comparison to a nodose ganglion from a MV2 case stained with mAb 12F10 (A) and mAb ICSM-18 (B).

**Fig. 1:**
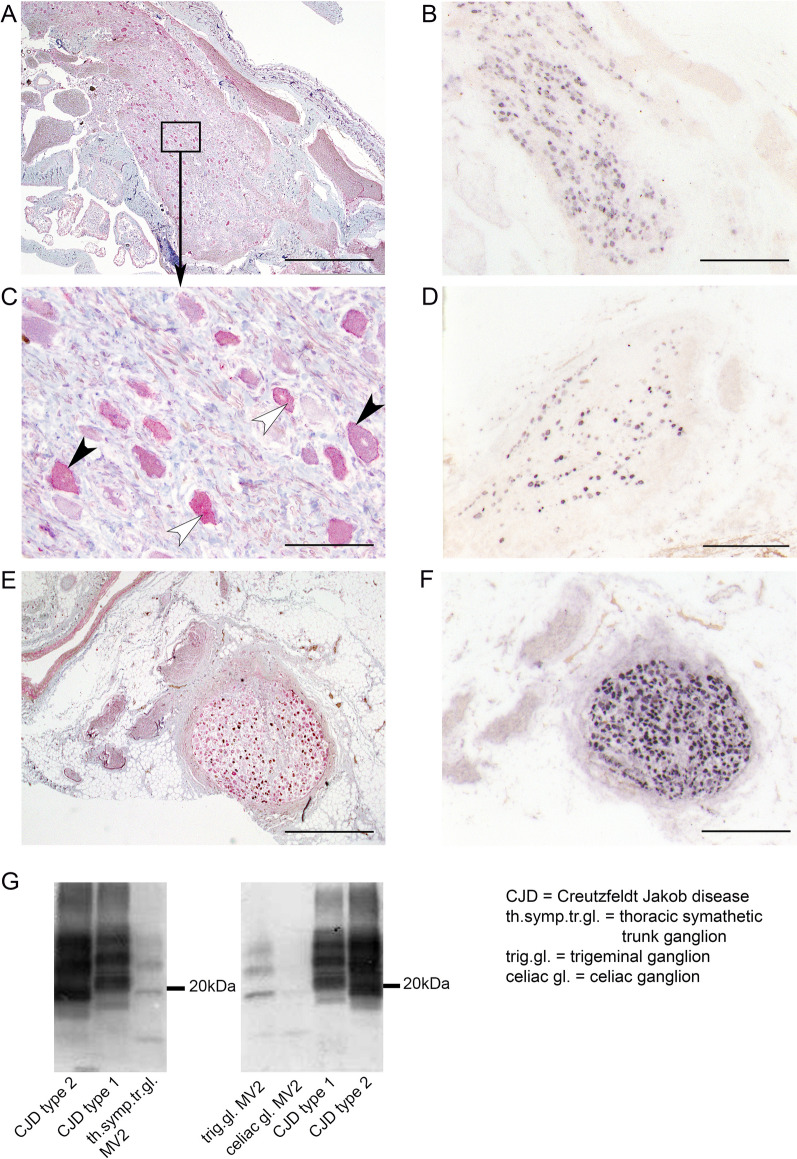
PrP^Sc^ aggregates in peripheral ganglia of sCJD patients detected by PET-blot and immunohistochemistry. Prion protein aggregates within several ganglia are visualized by conventional immunohistochemistry (**A**, **C**, **E**), and with the PET-blot technique (**B**, **D**, **F**). Membrane associated and cytoplasmic prion aggregates were detectable with both methods and are pointed out with arrowheads in black (membrane-associated) and white (cytoplasmic) in the area (**C**), magnified from A. Prion protein aggregates are shown in the trigeminal ganglion (**A**, **B**, **C**), nodose ganglion (**D**) and thoracic sympathetic trunk ganglion (**F**, **E**). Bars equal either 1 mm (**A**, **E**), 500 µm (**B**, **D**, **F**) or 50 µm (**C**). Western blot analysis (**G**) reveals the presence of CJD type 2 prion aggregates in a sympathetic trunk ganglion, a trigeminal ganglion and a celiac ganglion of a MV2 CJD patient. The typical three-banded pattern with a diglycosylated, monoglycolsylated and unglycosylated fragment are visible; the latter having a size of ~ 21 kDa in CJD type 1 and ~ 19 kDa in CJD type 2 brain homogenates (see CJD type 1 and 2). Immunodetection was performed using the monoclonal anti-prion antibody 3F4

### Immunohistochemistry

Tissue rehydration was followed by proteinase K digestion at a concentration of 20 µg/ml and a temperature of 37 °C for 30 min. After washing in TBS, heat-induced epitope retrieval was performed by boiling the slides in 4 mM HCl. Slides were rinsed in distilled water and TBS, denatured in 4 M GdnSCN and rinsed again, followed by a blocking step with 0.2% casein. The primary antibody 12F10 diluted 1:500 in TBS was added for 90 min at room temperature. Further rinsing with TBS was followed by incubation with the AP-linked secondary goat-anti-mouse antibody (DAKO) diluted 1:500 in TBS for 60 min at room temperature. New fuchsine (*Sigma-Aldrich*) was used to visualize the immunoreaction. The reaction was stopped with distilled water once the positive control had reached an adequate signal. Sections were lightly counterstained with hemalum (*Merck*) and differentiated with HCl-alcohol if necessary (150 ml isopropanol, 2 ml 30% HCl ad 500 ml with double distilled water).

### Evaluation

For statistical analysis of data we employed the *Pearson chi-square test of independence*. Pictures were taken using the *cellSens* software (*Olympus*). White balance and figure composition were carried out using *photoshop 21* software.

### Western blot

A 10% homogenate of native tissue in PBS with 0.5% DOC detergent (*Sigma-Aldrich*) was used for SDS-PAGE in 15% gels after a digestion step at 37 °C with proteinase K (50 µg/ml for 35 min) as described previously [37]. Gels were blotted onto nitrocellulose membranes (0.45 µm pore size, BioRad). We treated the membranes with 4 M GdnSCN (30 min) to achieve antigen retrieval and decontamination and blocked the membranes with casein 0.2% in TBS containing 1% Polysorbat before incubating them overnight at 4 °C with the primary antibody 3F4 (1:3000 in TBS with 0.02% casein). The horseradish peroxidase-coupled secondary antibody (Envision, *Dako*) diluted 1:1000 in TBS with 0.02% casein was added for 60 min and the antigen–antibody reaction was visualized on an x-ray film (Amersham Hyperfilm™ ECL, *GE Healthcare*) using chemiluminescence reagents (*Super Signal Femto West*).

## Results

### Detection of prion aggregates in ganglia of sCJD patients

Using the PET-blot method, prion protein aggregates were detected in 22 of the trigeminal ganglion samples (61%) (Fig. 1B) and in five of the nodose ganglia (31%) (Fig. 1D, Additional file 1: Fig. 1A, B). No protein aggregates could be found in any of the samples from the cervical sympathetic trunk or the stellate ganglion. However, we discovered prion protein aggregates with the PET-blot in three samples from the thoracic sympathetic trunk ganglia (27%) (Fig. 1F), in four samples from the celiac ganglia (11%), and in the ganglia of the lumbar sympathetic trunk in one patient (11%). No aggregates were detectable in the negative controls. Clearly affected cells directly neighboring ones that appeared to be free of pathologic aggregates were a common feature. The cellular characteristics of the prion protein deposits were found to be either membrane-associated or diffuse-cytoplasmic, which was confirmed by conventional immunohistochemistry (Fig. 1A, C). While prion protein aggregates were never detectable in samples of the vagus nerve, we did observe prion protein aggregates within axonal fibers that were presumably related to the trigeminal nerve of one trigeminal ganglion. PET-blot negative controls were always immunonegative for prion-specific antibodies in conventional immunohistochemistry (IHC).

For the trigeminal, nodose as well as the thoracic sympathetic trunk ganglia the positive PET-blot results could be confirmed by conventional immunohistochemistry (Fig. 1A, C, E). Here, the IHC staining intensity was comparable to the PET-blot staining for the trigeminal ganglia and the thoracic sympathetic trunk ganglia. In the nodose ganglion, IHC staining was present (not shown), but clearly weaker than the signal obtained with the PET-blot method (Fig. 1D). Within the celiac ganglia and the lumbar sympathetic trunk ganglion, prion aggregates could only be detected with the PET-blot method.

Using Western blot analysis, type 2 prion protein aggregates with an unglycosylated fragment of approximately 19 kDa were detectable in the trigeminal, celiac as well as thoracic sympathetic trunk ganglia as shown exemplarily for an MV2 patient in Fig. 1G. Prion protein aggregates could not be detected in the nodose ganglion. Samples of the cervical sympathetic trunk or the stellate ganglion were devoid of prion aggregates with this method as they were with PET-blot and IHC. The brain homogenate controls for CJD type 1 and type 2 displayed the usual three-banded pattern with diglycosylated, monoglycosylated and ungycosylated fragments; CJD type 1 displaying an unglycosylated fragment of 21 kDa and type 2 having a slightly smaller ungycosylated band of 19 kDa as described previously [28].

### Influence of prion type and codon 129 polymorphism on ganglia involvement

**Fig. 2:**
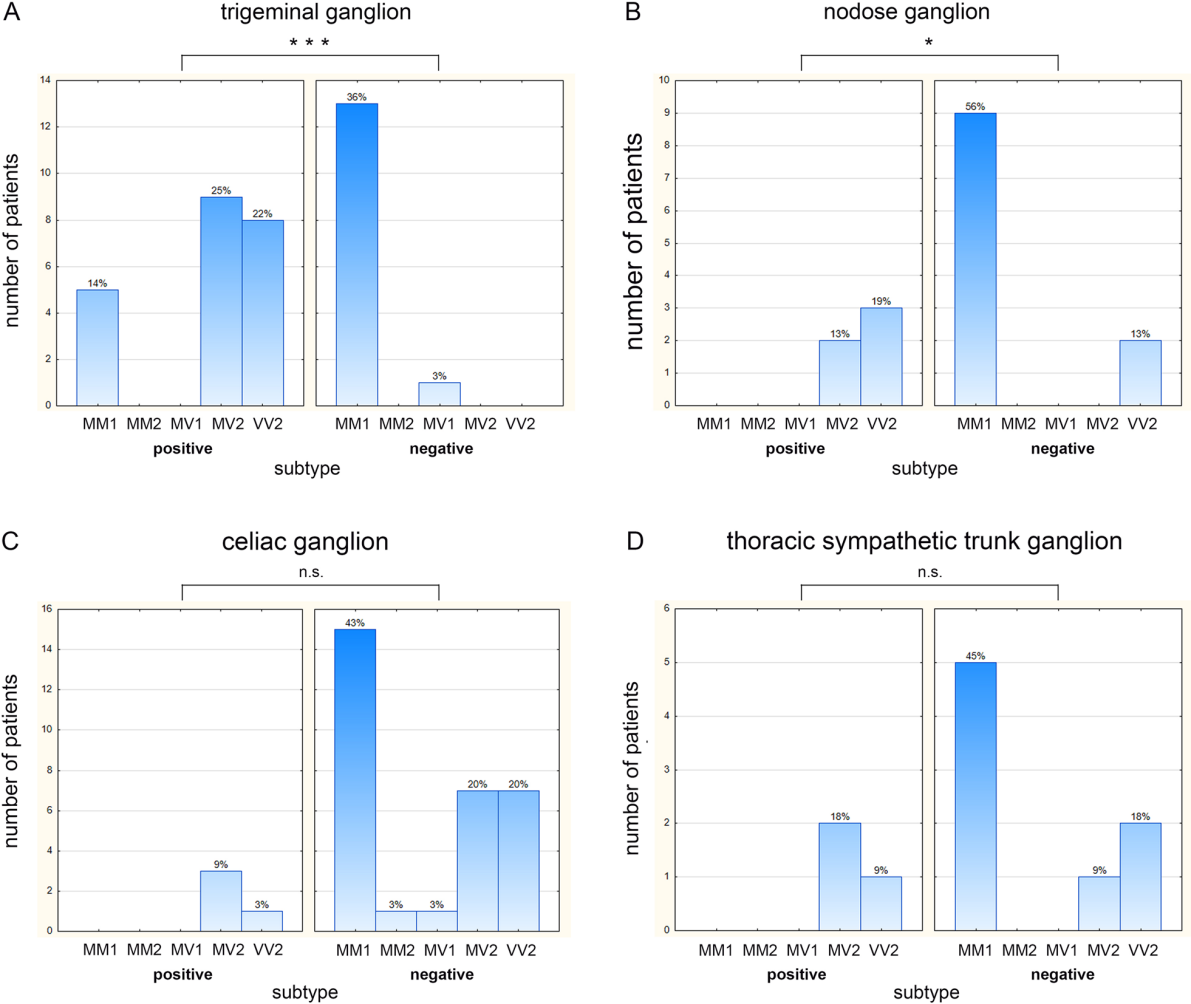
CJD subtype distribution in prion-accumulating and prion-negative peripheral ganglia. Within each figure (**A**–**D**) the left panel depicts the ganglia that stained positive for prion aggregates, while the right panel shows the ganglia that were tested negative. The abscissa displays the different subtypes of the prion-accumulating and not prion-accumulating ganglia. The ordinate gives the number of corresponding patients. The percentages indicate their respective portion in relation to all ganglia of a certain type (e.g. the trigeminal ganglion, **A**) investigated. Statistical analysis of ganglion involvement per disease subtype showed a highly significant association for the trigeminal (***p < 0.001) (**A**) and significant association for the nodose ganglion (*p < 0.05) (**B**), whereas no statistically significant correlations could be found for the thoracic sympathetic trunk or the celiac ganglia (n.s. *p* > 0.05) (**C**, **D**)

When stratifying the detectability of prion aggregates in ganglia according to the clinical and pathological disease phenotype, defined by the methionine/valine polymorphism at codon 129 of the prion protein gene in combination with the prion protein type 1 or 2, remarkable differences become apparent. Analysing the involvement of the trigeminal ganglia, which anatomically belongs to the dorsal root ganglia, prion protein aggregates were detectable in all MV2 and VV2 patients, five out of 18 MM1 patients (28%) but not in the MV1 patient. In four out of the five MM1 trigeminal ganglia, the intensity of the immunoreaction was week, whereas the immunoreaction in 10 out of 17 MV2/VV2 trigeminal ganglia was intense (see Table 1). The association between an involvement of the trigeminal ganglion and the underlying disease subtype was statistically significant (*p* < 0.001; Fig. 2A). In all autonomic ganglia that were investigated (nodose, stellate, cervical-, thoracic-, lumbal sympathethic and celiac ganglia), prion aggregates were never detectable in prion type 1 patients but in 30 out of 72 autonomic ganglia of prion type 2 patients. In the nodose ganglia, prion aggregates were detectable in both available MV2-, three out of five VV2- but mone of the nine MM1 ganglia. This is a statistically significant association (*p* < 0.05; Fig. 2B). While all investigated stellate and cervical ganglia were devoid of prion aggregates, in thoracal ganglia in none of five MM1, but in two out of three MV2 and one out of three VV2 ganglia, prion aggregates were detectable. In the celiac ganglia, in none of 13 MM1, not in the MV1, but in three out of 10 MV2 and one out of 8 VV2 ganglia, prion aggregates were detectable. In summary, the autonomous ganglia in which prion protein aggregates were detectable belonged to MV2 or VV2 patients, albeit a statistically significant association for this observation could not be demonstrated (Fig. 2D). The influence of the sCJD phenotype on the involvement of autonomous ganglia seems to be mainly explained by the prion type.

## Discussion

The results of this systematic neuropathological study show that ganglia of the autonomous nervous system may be involved in sporadic Creutzfeldt–Jakob disease. With the exception of the trigeminal ganglia, pathological prion protein aggregate depositions in peripheral ganglia were restricted to those patients that expressed type 2 prion aggregates during the course of their disease.

Previous studies have demonstrated an involvement of autonomous ganglia in vCJD [11, 22], but have generally failed to demonstrate such involvement in sporadic CJD [11, 12]. The only exception was the detection of prion protein deposits in the celiac ganglion of one sCJD patient [22] without characterization of prion type or codon 129 polymorphism. The reason for the inability to detect the involvement of peripheral ganglia in sCJD may be that only tissues of single subjects have been investigated so far and that the detection techniques may have lacked the necessary sensitivity. Our own results also show that while conventional immunohistochemistry could confirm the staining pattern and presence of prion aggregates in most ganglia, it could not detect prion aggregates in celiac and pelvic plexus ganglia. With the more sensitive PET-blot, however, the presence of distinct prion aggregates in these ganglia was evident. Of course, even with this sensitive method the possibility that in PET-blot negative ganglia prion aggregates exist below the detection threshold have to be taken into account.

The deposition pattern of protein aggregates, demonstrated in our study with the PET-blot method, is consistent with previous histomorphologic descriptions of affected ganglia [10, 11, 19]. Particularly Lee et al. also described the coexistence of membrane-linked and cytoplasmic deposits in human prion diseases [22].

As the physiological neuronal prion protein is necessary for the maintenance of peripheral myelin [6], and the spread of disease after peripheral uptake is mediated via the autonomous nervous system [24], an involvement of the peripheral nervous system in prion diseases is likely. Indeed, in some sporadic as well as in hereditary CJD, a demyelinating peripheral neuropathy has been reported [1, 2, 9, 18, 25, 26, 36, 42]. The aspect of demyelination indicates that the disease may cause a loss of function of the physiological prion protein in peripheral neurons by the conformational change of the prion protein leading to aggregates.

A detailed analysis revealed that only prion type 2 was detectable in peripheral nerves of CJD patients suffering from a peripheral neuropathy [2, 42]. This is in line with our findings, showing that a CNS-distant involvement of autonomous ganglia was only detectable in patients accumulating prion type 2.

### Why does a selection favoring prion type 2 occur?

An axonal spread may depend on the prion type, or the prion replication in peripheral tissues may be dependent on the prion type. A prion type describes the pathogenic agent in the original host, primarily characterized by its protein conformation. After transmission to a new host, different so-called prion strains can derive from it through interactions of the source prion type conformation with host-dependent modifiers, finally determining the clinical syndrome in the new host [38]. Collinge and colleagues described prion strains as an ‘ensemble’ consisting of one main component and, to a lesser extent, of several other forms [8]. Under the influence of the new host, selection pressure after transmission can lead to the propagation of a formerly underrepresented component of the prion `cloud´ [23], replacing the original predominant constituent. The simultaneous occurrence of prion types 1 and 2 in sCJD patients [29] fits in with the prion cloud hypothesis.

Meanwhile, it is known that the prion type concept is not restricted to human prion diseases but is also applicable to other species and has already been adopted to different forms of sheep scrapie [37] and BSE [41]. From animal experiments we know that after prion transmission a change of the donor prion type may occur in the host [33, 34]. Wemheuer and colleagues suggested a `selection of conformers´ in the lymphoreticular system of the gut, favoring type 2 replication [38]. Shikiya et al. reported that in PrP^Sc^ type 1 but not in PrP^Sc^ type 2, degradation outweighed prion formation in lymphoreticular tissues [32]. This is in line with the observation that prion diseases transmitted orally in herds of animals (classical scrapie, bovine spongiform encephalopathy, chronic wasting disease) are type 2 disorders [38]. Recent experiments on the peripheral spread of different prion strains in transgenic mice that were generated from sCJD also showed that the prion type 2 in combination with the valine polymorphism at codon 129 of the prion protein gene, derived from the sCJD subtypes VV2 and MV2, most effectively propagated in Ki-Hu129V/V and Ki-Hu129M/M mice after intraperitoneal inoculation [21].

The detection of prion protein type 2 aggregates in the celiac ganglia and ganglia of the sympathetic trunk in sCJD seems to be quite compatible with the hypothesis of prion type selection. In addition to a selection by prion type-dependent replication, a prion type-dependent property of axonal spread might be possible. This may explain why both prion protein types were detected when the spread takes place just over short distances, e.g. to dorsal root ganglia like the trigeminal ganglia. In contrast, only prion type 2 was found in more CNS-distant ganglia.

### Do the distribution patterns of detectable prion protein aggregates in peripheral ganglia tell us something about the direction of spread?

According to the route of prion spread after oral uptake [24] that has been confirmed for classical scrapie [35], classical BSE [16] and chronic wasting disease in deer, the involvement of trigeminal ganglia, nodose ganglia as well as thoracic and lumbar sympathetic ganglia is consistent with a centrifugal spread from the central nervous system to peripheral ganglia.

An involvement of the celiac ganglia was seen in the hamster model of oral challenge as the next step after the enteric nervous system involvment [24]. We were able to detect prion aggregates in the celiac ganglion in three out of nine MV2 and one out of eight VV2 patients (Table 1). This opens the possibility of a centripetal spread according to the model of oral challenge [24].

In sympathetic ganglia of the studied patients, the cervical and stellate ganglia were consistently negative, whereas prion aggregates were detectable in single thoracic and lumbar ganglia of prion type 2 patients. In an orally BSE-challenged Cynomolgus monkey model, Holznagel et al. detected prion accumulation earlier in the root entry zone of lumbar spinal cord segments and dorsal root ganglia, as in the ventral horn, the intermedio-lateral column or the sympathetic ganglia [17]. In both the scrapie and the monkey model, prion aggregates were found at first in the gastrointestinal lymphatic and nervous system after oral uptake [17, 24]. The investigation of the gastrointestinal system was not in the scope of the present study. Although the data from our study do not indicate a centripetal prion spread, an investigation of the gastrointestinal lymphatic and nervous system would be necessary to definitively exclude this.

## Conclusion

By using the sensitive PET-blot method for protein aggregate detection, we were able to show that ganglia are involved in the pathological process of prion aggregation in sporadic Creutzfeldt Jakob disease. Whereas aggregates of both prion types may spread to dorsal root ganglia, more CNS-distant ganglia seem to be only involved in patients accumulating prion type 2. The prion type 2 selection involving CNS-distant ganglia may be due to prion type-dependent replication, or a prion type-dependent property of axonal spread. In analogy to animal models of oral challenge, the involvement of autonomous ganglia in the pathophysiological process of Creutzfeldt–Jakob disease raises the question, whether some of so-called "sporadic" Creutzfeldt–Jakob diseases may be acquired. Our data are consistent with a centrifugal spread from the CNS to autonomous ganglia. However, to rule out a centripetal spread as a sign of disease acquisition, additional investigations of peripheral tissues that allow a comparison to animal models of oral challenge will be necessary.

## Supplementary Information

**Additional file 1: Figure 1**: PET-blot analysis of a nodose ganglion from a MV2 patient stained with the mAb 12F10 (A) and ICSM18 (B) and a negative control (AD patient) stained with mAb 12F10. Bar = 500 µm (JPG 1271 kb)

## Data Availability

The data are presented in tables and figures of the article. Materials used in the current study are available from the corresponding author on reasonable request.

## Abbreviations

BSE: Bovine spongiform encephalopathy
CJD: Creutzfeldt–Jakob disease
CNS: Central nervous system
dpi: Days post infection
GdnSCN: Guanidine thiocyanate
IHC: Immunohistochemistry
MM1/MM2: Methionine homocygous at codon 129 of the prion protein gene in combination with prion protein type 1 or 2
MV1/MV2: Methionine /valine heterocygous at codon 129 of the prion protein gene in combination with prion protein type 1 or 2
PET-blot: Paraffin-embedded tissue blot
*PRNP*: Prion protein gene
PrP^C^: Prion protein cellular = physiological form
PrP^Sc^: Prion protein scrapie = misfolded form
sCJD: Sporadic Creutzfeldt–Jakob disease
vCJD: Variant Creutzfeldt–Jakob disease
VV2: Methionine homocygous at codon 129 of the prion protein gene in combination with prion protein type 2
