# Supplementary figures and images for: Prion type 2 selection in sporadic Creutzfeldt–Jakob disease affecting peripheral ganglia
Source: Acta Neuropathol Commun. 2021 Nov 24;9:187. doi: 10.1186/s40478-021-01286-4 (PMC8611978; doi:10.1186/s40478-021-01286-4)

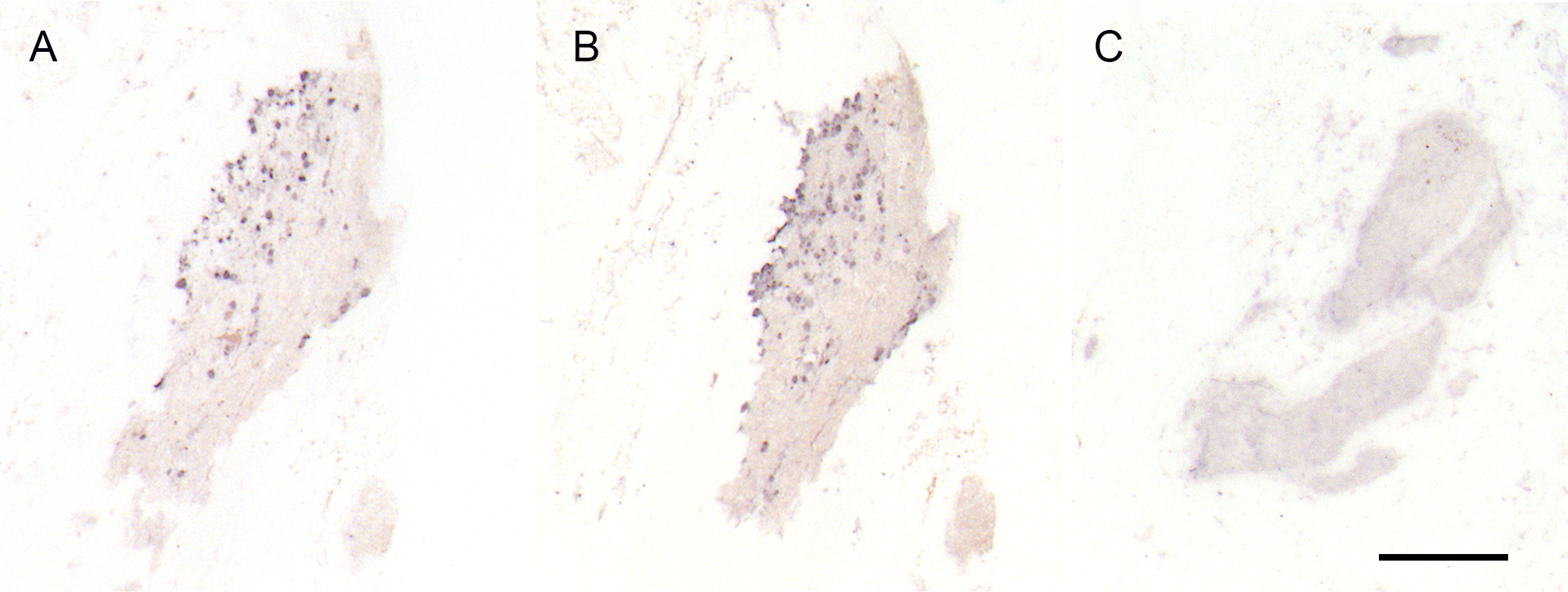

Supplement: Supplementary file 1 — Additional file 1: Figure 1: PET-blot analysis of a nodose ganglion from a MV2 patient stained with the mAb 12F10 (A) and ICSM18 (B) and a negative control (AD patient) stained with mAb 12F10. Bar = 500 µm (JPG 1271 kb) [file 40478_2021_1286_MOESM1_ESM.jpg]
